# Safeguarding Reimagined: Centering Athletes’ Rights and Repositioning Para Sport to Chart a New Path

**DOI:** 10.3389/fpsyg.2022.815038

**Published:** 2022-05-03

**Authors:** Yetsa A. Tuakli-Wosornu, Sandra L. Kirby

**Affiliations:** ^1^Department of Social and Behavioral Sciences, Yale School of Public Health, New Haven, CT, United States; ^2^Department of Physical Medicine and Rehabilitation, University of Pittsburgh, Pittsburgh, PA, United States; ^3^Faculty of Graduate Studies, University of Winnipeg, Winnipeg, MB, Canada

**Keywords:** safeguarding, human rights, violence prevention, harassment and abuse, Paralympic sport, athletes

## Abstract

**Objectives:**

Para sport has much to teach the broader sports world about safeguarding and athlete protections. By centering athletes’ human rights and underlining the rights-based philosophical underpinnings of the Paralympic Movement, we outline how sport can be safer to all players, coaches, and other participants.

**Methods:**

We address global Human Rights conventions and their application to Para and non-disabled sport. Safe Sport is positioned as a matter of human rights. The nature of interpersonal violence that human beings experience within and outside sport is discussed. The intersectionality of vulnerable identities (related to gender, sexuality, disability, ethnicity, etc.) is reviewed in some detail.

**Results:**

Rights violations in Para and non-disabled sport illustrate both individual and organizational vulnerabilities. Individual- and organizational-level drivers of abuse, as well as various modes and types of abuse observed in Para sport, are relevant in all sport settings and should be centered in global sport safeguarding work. The rights-based core of Para and similar sports movements, exemplifies this.

**Conclusion:**

From a Para-informed vantage point, we issue a call to action, where interpersonal violence in sport is reduced by leveraging relevant elements of the Paralympic Movement. This call asks all sport participants to reject a purely capitalist approach to sport and follow a Para sport paradigm; which embodies human achievement (including sporting success), reflects human rights and inherent human dignity, and requires a higher standard of behaviour.

“When you go out to paint, try to forget what objects you have before you… Paint it just as it looks to you, the exact color and shape, until it gives you *your own impression* of the scene before you” *-Oscar-Claude Monet.*

## Introduction

Global sports organizations are approaching safeguarding and athlete protections with increased focus and rigor (Mountjoy et al., 2016; Vertommen et al., 2016; Kerr et al., 2019a; Hartill et al., 2021; Rhind et al., 2021; Rutland et al., 2022). Many scientific and advocacy teams acknowledge that safeguarding is a systems-level, rights-based issue that requires culture change (Lang, 2020; Tuakli-Wosornu et al., 2020; Campbell and Tincknell-Smith, 2021; Komaki and Tuakli-Wosornu, 2021; Tuakli-Wosornu, 2021). Nevertheless, sport safeguarding methods and efforts tend to rely on ableist, Western, heteronormative, and otherwise majority-centered perspectives—as do the data informing those efforts, which limits their generalizability to diverse sport contexts (Brackenridge et al., 2010a). Furthermore, it is unclear how successful traditional safeguarding approaches have been in genuinely improving athletes’ safety and wellbeing in sport. There may be value in considering a fresh point of view.

Anchored in the human rights of athletes, the Paralympic Movement offers a deeper understanding of what sport can be to players, coaches, fans, and all sport actors (Bailey, 2008).

## Objectives

The aim of this paper is to underline the rights-based philosophical underpinnings of the Movement, center a Para sport perspective, and offer a new impression of how to deliver safe, inclusive, and joyful sport environments for all.

## Materials and Methods

The authors first review global Human Rights conventions. Their application to Para and non-disabled sport are then reviewed and anchored within sport safeguarding. Next, the rights-based foundation of the Paralympic Movement is examined utilizing a literature review and relevant document analysis. Finally, a safeguarding lens is used to pinpoint the nature of interpersonal violence human beings experience in sport and what specifically is known about the nature and scope of interpersonal violence within Para sport. Ultimately, the analysis suggests that Para and similar sport movements have much to teach the sports world about athlete protections, and may naturally adopt leadership positions in this space.

## Results

### Anchoring Athlete Safeguarding/Protections in Human Rights

All sport contains the potential for athlete victimization. Athlete protections involve diverse children and adults, participating in a range of sports, in different ways, over time (Bekker and Clark, 2016). The consequences of interpersonal violence against and between athletes, as well as failure to protect athletes from harm, affects individual athletes, their families, their communities, and the health of sport and society at large (Brackenridge et al., 2001; Mountjoy et al., 2016; Vertommen, 2018; Vertommen and Parent, 2020; Fortier et al., 2020; Guiora, 2020; Tuakli-Wosornu et al., 2020; Tuakli-Wosornu, 2021; Rutland et al., 2022). Brackenridge has long argued that sport embodies a cultural island, set apart from and mostly revered by the rest of society. This has led to an “historic institutional blindness of sport to child abuses…[and] an almost complete absence of prevention measures (Brackenridge, 2017)”. It has really only been within the last three decades that sexual abuse scandals coupled with focused research have launched a field of study called safeguarding in sport or child/adult athlete protections. Central to the field is the idea that players “sit at the intersection between sport and human rights (Uni Global Union, 2017),” and have a right to play freely, undisturbed by antagonism and exploitation (Mountjoy et al., 2016). As the field of safeguarding holds athletes’ rights at its center, in each section of this paper, we address in order, the human rights perspective on safeguarding, followed by perspectives for Para and non-disabled sport (Campbell and Tincknell-Smith, 2021).

#### The Human Rights Context

Sport ‘done well’ can be one of the greatest global promoters of human rights (International Olympic Committee, 2020; Tuakli-Wosornu et al., 2021). The Olympic Charter underlines this point by stating, “the practice of sport is a human right. Every individual must have the possibility of practicing sport, without discrimination of any kind … with respect for universal fundamental ethical principles … and the preservation of human dignity (Mountjoy et al., 2017; International Olympic Committee, 2020).” All who participate in sport have the right to feel and be safe and be treated with dignity and respect. For Para participants, this is as much about equitable access and integration into sport settings as it is about safety.

Paolo David, former Secretary of the Committee on the Rights of the Child in the Office of the United Nations High Commissioner for Human Rights, described in 1998 and again in 2004 how the active protection of the human rights of athletes is critical to both the elimination of discrimination and violence in sport, and the future development of sport generally (David, 1998, 2004). A driving research question, somewhat avant-garde at the time, was “Can the integration of human rights in the sport system improve its quality, and the status of athletes, including its youngest ones? (Brackenridge, 2006).” This re-positioned an ethic of holistic athlete care and whole-person development as the true cornerstone of sport; calling out the ethical risks of disconnecting and distancing an athlete’s physical fitness and performance goals (intrinsic or imposed) from their personal agency (real or perceived) as they act within the realm of sport (David, 2004; Brackenridge, 2006). That is, the effective “promotion and protection [of] the health and well-being of young people in sport requires some understanding of human rights in general… (Kirby and Demers, 2014).”

The *United Nations Universal Declaration of Human Rights* (United Nations, 1948) recognizes the “inherent dignity and the equal and inalienable rights of all members of the human family (which is) the foundation of freedom, justice and peace in the world (United Nations, 1948).” United Nations’ (UN) conventions are progressive, moving from the original human rights Declaration (1948) which mandates the elimination of discrimination in various forms, to articulating protections and entitlements that must be afforded specific populations including children, indigenous peoples, persons with disabilities and others.

The *United Nations Convention on the Rights of the Child* (UNCRC, 1989) recognizes that children have rights and that children may need ‘special safeguards and care as necessary for his or her well-being (Preamble) … and to reach their fullest potential’ (Article 29; UNCRC, 1989). This includes the rights of children to “rest and leisure, to engage in play and recreational activities” (Article 31). Since many children, including those with disabilities, participate in sport around the world, the UNCRC is an excellent guide for such participants, and those responsible for them, to understand and respect those rights.

For Sport, the *United Nations Convention on the Rights of Persons with Disabilities* (UNCRPD, 2007) guarantees that each child is born free and equal in dignity and due merits (UNCRPD, 2007). This convention reflects a shift to seeing persons with disabilities as having indivisible human rights, as being equal members of society, and by extension, as Para athletes with equal claim to full participation in the world of physical activity and sport.

#### The Sport Context

Sport reflects elements of society. As such, there is no reason to believe that sport is exemp from child and adult rights violations, and should be excluded from scrutiny. During the last decades, concerns for child athletes have included the high risk of excessively intensive physical training; psychological, physical and/or sexual abuse, neglect; violence on and off the field of play; doping; economic exploitation; displacement; trafficking and sale (e.g., underage athlete contracts); transfers and reduction of freedom of association; limits to the right to education; and limits to civil rights and freedoms of athletes (David, 1998, 2004; Mountjoy et al., 2017). Once a child is labelled an athlete, “it is frequently the case that their identity as children first is lost and their rights as children are eroded (Mountjoy et al., 2017).”

Sport is a full range of organized physical activities from local recreational participation to international multi-sport competition. Sport can be played for itself or used as a tool, as for example, for health, global community development and/or for re-building traumatized communities (Coalter, 2007; Beutler, 2008; Mountjoy, 2011; Hatzigeorgiadis et al., 2013; UNESCO, 2015). Children and adults participate in sport by the millions around the world. When sport is ‘done well,’ participants learn values such as equality, fairness, sharing and striving to achieve with others (International Olympic Committee, 2020). These values cross genders, races, ethnicities, ages, sexual identities and physical abilities (UNESCO, 2015). Sport is however, also a global entity which, in its various organizational forms, sets international competitive standards and practices and coordinates World, Olympic and Paralympic Games. These opportunities for participation/competition and rules of practice, set at the most elite levels, are largely replicated in and supported by national and regional sport practices.

A feature of organized sport is that it remains largely independent in how it manages its business and finances; how it conducts its programs, games and competitions and even how it prescribes expected behaviours. Rules are made and when rules are broken, organized sport has largely policed itself. The International Paralympic Committee (IPC), for example, has an established a Code of Ethics which prescribes what is acceptable in Para sport (IPC Code of Ethics, 2016). Disputes or disagreements between parties may be resolved by an internal committee, or even at the highest level, with The Court of Arbitration for Sport (CAS; Court of Arbitration for Sport, 2020).

With the UN conventions to theoretically protect the general public from violence, and on the heels ot the UN World Report on Violence against Children, UNICEF supported research to protect the world’s children from violence in sport (Pinheiro, 2006; Brackenridge et al., 2010a). This culminated in an invited report by Brackenridge and Fasting (2009), a follow-up to Pinheiro’s study with a focus on sport that put the issue of violence against children in sport firmly on the world map (Brackenridge et al., 2010a). Concurrently, rights advocates and researchers in sport were able to work directly with the International Olympic Committee (IOC) on the Consensus Statement on Sexual Harassment and Abuse (IOC, 2007); revised in 2016 (Ljungqvist et al., 2008; Mountjoy et al., 2016). There was some focus on athletes with disabilities in each of the IOC meetings, and some of that information made it into the final consensus statements. With those and the legacy of the 2012 Olympic and Paralympic Games, the IOC had its first “Safeguarding Team” present at the 2016 Rio Olympic Games. In the same year, the IPC revised and expanded its policy on the prevention of sexual harassment and abuse in Para sport. The updated statute reflected a more comprehensive acknowledgement of the full range of modalities and contexts of interpersonal violence in Para sport, beyond sexual harm alone (IPC, 2016a). Consistent with similar efforts at the IOC, the IPC implemented a Games-time Safeguarding Office, and appointed one of the authors (then an IPC medical committee member) as the inaugural Games-time Welfare Officer at the 2016 Rio Paralympic Games. UNICEF published a set of International Standards for Safeguarding and Protecting Children in Sport and later the IOC Tool Kit was published to help International Sports Federations protect their athletes (IOC, 2017; Rhind and Owusu-Sekyere, 2017). Work is currently ongoing with many of the International Federations (ASOIF, summer sports and GAISF, winter sports) as they are variously bringing safeguarding practices to their international events and to their member federations.

As the evidence of interpersonal violence against athletes has grown, the field of study has globalized. It now includes international education programs, policies and practices, training for national and international federations, outreach to child’s rights and human rights experts, the emergence of survivor groups, the development of ‘safeguarding officers’ for every sport organization and most recently, the establishment of research hubs (e.g., Centre for Child Protection and Safeguarding in Sport, United Kingdom; Gender Equity in Sport Research Hub, Canada) for the collection, synthesizing and rapid distribution of information (Damjanovic, 2020). International and some national broadcasters have been valuable in carrying safeguarding information forward to all (Cohen, 2020; Spooner, 2021).

In some nations, non-sport child protection organizations are reaching into sport. Two excellent examples of such partnerships are the Canadian Centre for Child Protection (C3P) and Child Protection in Sport Unit (CPSU; Child Protection and Sport Unit, n.d.; Canadian Centre for Child Protection, n.d.). Both have some focus on Para participants. The Commit to Kids framework is used electively across all levels of sport, to help mitigate the risk of child sexual abuse within organizations. Special emphasis is placed on children with disabilities and the principles of universal design. In the United Kingdom, the Child Protection in Sport Unit (NSPCC-CSPU) partners with Sport England, Sport Northern Ireland and Sport Wales to work with the sport governing bodies to reduce child abuse. Of note, the CPSU has extensive resources for deaf and disabled children, and young people (281 documents). Their work emphasizes appropriately responding to concerns, breaking down barriers, anti-bullying, coaching advice, social inclusion, and inclusive coaching.

Very recently, attention is being paid to other actors in sport such as adult athletes, coaches, attention is being paid to other actors in sport such as adult athletes, coaches, administrators, referees, parents and medical personnel. All who participate in sport should benefit from protections offered.

#### The Para Sport Context

A note on terminology. There are strengths, as well as stubborn controversies embedded in decisions to use person-first language, where a personhood-noun is followed by the description of a disability (i.e., ‘athletes with disabilities’), versus identity-first language, where disability plays the role of adjective, and precedes the personhood-noun (i.e., ‘disabled athletes’; La Forge, 1991; Wehmeyer et al., 2000; Gernsbacher, 2017). Acknowledging the debate, but finding merit in both these language strategies, the authors have decided to alternate between two otherwise conflicting linguistic rules throughout this manuscript (as above and below; Wehmeyer et al., 2000; Vaughan, 2009; Collierm, 2012; Andrews et al., 2013; Smith, 2016; Crocker and Smith, 2019).^1^

Similarly, language used to describe adaptive sports has evolved over time. While antiquated terms such as ‘handicapped sport’ have all but disappeared from modern literature, the term *Para* (capital P, space, no hyphen) was put forth in 2016 by the IPC and refers to non-Paralympic Games (i.e., non-Games-time) events or activities that are under the jurisdiction of the IPC or an IPC member and are governed by the requirements of the IPC Classification Code (IPC, 2016b,c). We primarily use this term. *Paralympic*, *Paralympics* and *Paralympian* can only be used with reference to the Paralympic Games. For all sport outside of that, the word Para can be used (capitalized and followed by a space), provided that the International Federation (IF) is a member of the IPC or recognized by the IPC. Para is not just used for events but also athletes, e.g.

Para Triathlete, provided that the IF is a member of the IPC or recognized by the IPC. In this manuscript, we occasionally use *Paralympic*, *Paralympics* and *Paralympian* where appropriate.

There are several streams of Para athletes and much diversity within those streams (Kirby et al., 2008; Vertommen et al., 2016; Tuakli-Wosornu et al., 2020; Rutland et al., 2022). There are at least three categories in which participants compete globally: the visually impaired, those with physical impairment and those with intellectual challenges (IPC, 2016c). The *International* Committee of *Sports* for the *Deaf* (ICSD) is recognized by the IOC as a sports organization, and the word Para is not used for the deaf (International Committee of Sports for the Deaf, n.d.). The pinnacle event for those who are deaf is the Deaflympics (formerly the World Games for the Deaf). For those with physical, visual, and intellectual impairments, top level competition is the Paralympic Games (Gold and Gold, 2007). For those with intellectual impairments, there are also the Special Olympics, the Trisome Games (for athletes with Down syndrome), and the Global Games (organized by Virtus World Intellectual Impairment Sport, n.d.).

That disability is still defined as a comparison of Para athletes to those who are non-disabled remains problematic (Purdue and Howe, 2012). Para and Paralympic sport participation and performance, as some would argue, have expanded from recreational tournaments to include elite talent spectacles, as events like the Paralympic Games now follow the quadrennial pattern established by the IOC (Gold and Gold, 2007; Bailey, 2008; Howe, 2008). Yet and still, the IOC and the IPC are separate but unequal entities. The IPC is the capstone organization of the modern phenomena of the rapidly growing world of sport for those with physical, visual, and intellectual impairment (IPC, 2016; van Dijk et al., 2017; Tuakli-Wosornu et al., 2019). There are multisport Paralympic Games every 4 years, winter and summer, and athletes participate by sport and classification (Tuakli-Wosornu et al., 2019). Sometimes, equipment, technical, and physical supports are necessary to enable athletes to practice and compete.

Uniquely, Para sport federations have evolving rules to guide fair selection into sport classes, sport participation, sport competition, and medal events when referring to major championships and major Games – but these change intermittently, often not without controversy and real athlete-level impacts (MacInnes, 2020). The IPC and the IFs theoretically oversee that equal rights, a level playing field and adherence to the stated values of courage, determination, equality and inspiration are part of every Para athlete’s experience at the Paralympic Games and lesser competitions. However, these ideals can be threatened by unethical cheating behaviours such as the use of performance-enhancing medications under the guise of therapy or the intentional induction of autonomic dysreflexia, a phenomenon known as ‘boosting,’ in spinally injured athletes (McNamee and Parnell, 2018; Tuakli-Wosornu et al., 2019). It can also be threatened by IFs that may not intentionally or unintentionally comply with the IPC Classification Code, i.e., admitting persons who are not eligible to participate.

The logistics of the Paralympic Games are different in substantial ways from those of the Olympic Games, including the use of classifiers, limits to advertisements, the number, type and scoring of events and the provision and scheduling of supports to enable Paralympians to compete. For example, at the Paralympic Games where one of the authors attended as a World Rowing (FISA) umpire, volunteers were assigned to individual Paralympians to take care of shoes, wheelchairs, prothesis or whatever they left on the dock. This was not about housekeeping, but about ensuring that each athlete received their personal and necessary equipment when they exited from the water. The regard for the well-being and safety of athletes is both evident and paramount.

Despite the large percentage of persons with disabilities globally, opportunities for equal participation in all facets of society—including physical activity and sport, are still wanting (Swartz et al., 2016; Howe, 2019). This phenomenon is often worse in lower resourced areas, which are the places and spaces the overwhelming majority of global disabled persons call home (World Health Organization, 2011). If societal barriers are in place, children and adults with disabilities may be unable to participate fully and safely in Para sport. Outside sport, children and adults with disabilities are approximately four times as likely to be victims of interpersonal violence than the population as a whole (Sobsey and Doe, 1991; Sobsey, 1994; Jones et al., 2012). Those with intellectual impairment are at highest risk (Sobsey and Doe, 1991). As Mountjoy et al. write, “the human rights approach to sport indicates that consideration be given to the age, the gender and the disability of children in sport (Mountjoy et al., 2017).” Early statistics confirm that children with disabilities are not immune to interpersonal violence in sport (Vertommen et al., 2016; Kerr et al., 2019a; Tuakli-Wosornu et al., 2020; Rutland et al., 2022). Many are at risk in both society and sport.

The world of sport has made definite inroads here and appears aware of both the necessity for and challenges of incorporating the needs of persons living with disabilities into organized sport. The involvement of the IOC, IPC, UNICEF, Safe Sport International (SSI) and the many international and national federations are all examples of global efforts where sport researchers have come together with those working in human rights and on development to address violence against participants in both Para and non-disabled sports competitions.

### Philosophical Underpinnings of the Paralympic Movement

Centered on tenets of mutual understanding, fair play, and teamwork, sport has long been an agent of positive social change and a celebration of humanity. Yet unique amongst sporting initiatives, the Paralympic Movement takes as its point of departure a specific set of rights-based values, including respect for human dignity, acknowledgement of universal human rights, and emphasis on inherent human value (Bailey, 2008; Brittain, 2012; Legg, 2018). Identical values are central to athlete safeguarding – raising the question: should, how might, and where can Para sport take an active leadership role in the safeguarding conversation?

#### Paralympic Foundations

The Paralympic Movement was forged in war. Dr. (later Sir) Ludwig Guttmann, a skilled neurosurgeon and Jewish refugee fleeing Nazi Germany, is widely credited as the founder of competitive global disability sport (Hughes, 1999; Brittain, 2012). Guttman saw the disabled as valuable and respected members of the human community and this drove him to reach for a new vision of what disabled people could do and be – and the role sport could play. Revolutionizing the way paralysed veterans were rehabilitated and understood, he created an intensive, dynamic program culminating in the Stoke Mandeville Games (Guttmann, 1967; Goodman, 1986). Whilst few sporting movements are as deeply steeped in rights-leaning philosophies as the Paralympic Movement, it remains unclear whether safeguarding advocates are leveraging and/or looking to Para sport to enhance global athlete safeguarding in diverse sport settings effectively. A question (and challenge) the authors pose, is: if the Paralympic Movement is best suited to model respect for participants’ human rights, how can safeguarding advocates better distill its essential tenets to create positive change in global sport?

#### Paralympians Positioned as Separate and ‘Different’

While the Olympic Games have been around for longer than a century, the Stoke Mandeville Games did not become the Paralympic Games until 1960 in Rome, Italy (Brittain, 2012; Legg, 2018). The Paralympic Games were intended to function in parallel with (i.e., on equal footing and with the same global standing as) the Olympic Games, hence the term ‘Para’ (Hughes, 1999).

Through the lens of Critical Disability Studies, where institutions rather than impairments are understood to systematically disable people, the media can be considered an agent of disability apartheid (e.g., ‘special Games’ for ‘special athletes’) and, an echo chamber for hegemonic, ableist narratives that position Paralympians as sports people who by virtue of their participation (not athleticism or performance) have conquered disability (Goggin and Newell, 2000; Shildrick, 2012; Goodley, 2013). Some scholars go so far as to argue that the Paralympic Games itself – by virtue of being separate – is complicit (Goggin and Newell, 2000). Central to common media narratives about Para athletes are “stock stereotypes of ‘brave, elite athletes,’ ‘special people,’ ‘remarkable achievers’” and the unambiguous positioning of disability as a weakness, deficiency, or negative condition one should naturally aim to ‘beat (Goggin and Newell, 2000).’

The centering of disability (not athleticism or performance) in the relatively miniscule cannon of mainstream media stories about Paralympic sport has the very real effect of removing or lessening the general public’s perception that elite Para sport is about win-at-all-costs performance – but this may at the same time inadvertently remove or lessen the perception that elite Para sport is ‘elite’ (i.e., highly competitive, skill- and performance-based) at all (Purdue and Howe, 2012). When participation, social inclusion through sport, and overcoming disability become the focus rather than performance, assumptions afforded Olympians which contribute to the hypnotic nature of high-performance sport, fade away. These include highly competitive selection processes, hard-earned pride of place, and self-actualization through rigorous preparation.

Furthermore, a significant percentage of published scholarship concerning internationally competitive Para and Paralympic athletes analyses ‘sport’s role in eliminating social and institutional barriers and promoting inclusion,’ rather than injury prevention, mental skill, or physical performance (Howe and Silva, 2018; Kamberidou et al., 2019). This positioning is controversial among sport scientists (Howe, 2019). Further, there is no data to suggest that the Paralympic Movement does in fact measurably reduce disability stigma in the general population (McNamee and Parnell, 2018). Some argue that centering abstract, sentimental elements of sport like inspiration reaffirms the patronizing lens through which individual Para athletes and the Paralympic Movement generally tend to be viewed. This may be part of the reason why Para sports receive comparatively little attention in sports-related scholarship, even in high-priority areas such as longitudinal studies on athletes´ health -- including patterns of unintentional injury, illness, and intentional injury and violence (i.e., abuse), which has been identified as one of the biggest existential threats to modern sport (Finch et al., 2017; Hoy, 2019; Hirschmüller et al., 2020).

A new definition of “Paralympism,” the underpinning philosophy of the Paralympic Movement, has recently been put forth by McNamee and Parnell. In their paper, they assert that the Paralympic Movement is in many ways, “a celebration of sporting difference (McNamee and Parnell, 2018).” Being clear on differences and distinctions does seem a central and essential part of the Paralympic Movement’s philosophical identity. Athlete classification lies at the heart of the Paralympic Games – and this process is about the identifying fundamental differences between athletes and categorizing them into separate sport classes accordingly, rather than looking for similarities (Tweedy et al., 2014). Sport classification has been long debated in both Para and non-disabled sport, and issues surrounding modern gender non-binary athletes have recently brought this topic back to the fore in the Olympic Movement (curiously, the Paralympic Movement has heretofore been relatively mum on this topic; Karkazis and Jordan-Young, 2018; Sőnksen et al., 2018; Rizzone, 2022). Thus the idea that athletes’ differences matter in some way is found in all sport settings. Still, it must be acknowledged that the ever-evolving, resource-intensive, biopsychosocial classification system on which Para sport rests, is far and away one of the most complex *and* elemental phenomena in sport (Tweedy et al., 2014).

#### Paralympians – Internalized Difference

The active centering and even “celebration” of difference shows up in other ways and emanates from even deeper levels within the modern Paralympic Movement. While it is true that external forces such as the media and general public tend to regard Para athletes, actors, and achievements as ‘different’ than those in non-disabled sport, this tendency is apparently also internalized – Para sport actors themselves consistently separate themselves from Olympic groups, and “do their own thing,” intentionally.

From articulating a different set of sport values (courage, determination, equality and inspiration, rather than excellence, friendship and respect), to adopting a different set of rules of engagement, and even to responding differently to international sport crises, the Paralympic Movement sets itself apart from its Olympic counterpart (Myre, 2016; McNamee and Parnell, 2018). We neither endorse nor condone this trend, we are simply noting it. Looking ahead and recognizing that athlete safeguarding is an urgent matter of universal importance to all sports and that Para athletes arguably represent the archetypal group of concern, a few questions surface: how and when does separation serve the Paralympic and Olympic Movements?; is there anything to be gained by forging new alliances across sports movements, based on common goals and calls-to-action, by leveraging the ethos of Para sport in the service of universal safeguarding?; finally, is this possible/practical? Certainly, no sports movement should be absorbed into another. But with some vision, it may be possible to positively (and more efficiently) neutralize toxic sport cultures through shared resources (philosophical, technical, human, and other) and new approaches. We see this even now, as the IPC and IOC increasingly cooperate and interact with staff, board, and commission representation, as well as Games’ delivery.

### Vulnerability to Rights Violations and Intersectionality: Human Rights, Sport, and Para Sport

#### Who Is Vulnerable to Human Rights Violations?

As we shine a light on ending abuse against those who participate in sport, we necessarily first turn our attention to victims of interpersonal violence. The core human rights treaties described in section “The Human Rights Context” present, in one way, a blueprint for sport as it moves to provide safe opportunities for all to the benefits of physical, social, and psychological health and development. Victims of interpersonal violence, including those in sport, are likely to be discriminated against of the basis of some combination of sex, gender, race, age, occupation, economic status, disability, social or cultural minority status, and/or live in environments ridden with conflict/war, or as migrants/refugees (Cooper, 2016; Mountjoy et al., 2016; Tuakli-Wosornu, 2021). Sport, as part of society, ensures that these discriminations exist within it.

Reviewing the work of Pinheiro who focused on the threats to children, using an ecological model for understanding risk and protective factors for interpersonal violence: Society ➔ Community ➔ Relationship ➔ Individual (Pinheiro, 2006). He illustrated that a combination of factors is at work to influence when abuse will occur, reoccur, or stop. The human rights list of who is vulnerable to violence is lengthy. While Pinheiro’s model addresses interpersonal violence against children, it could easily be expanded to interpersonal violence against adults. Sport is one of many sociocultural contexts in which children and adults interact fluidly in (interpersonal) relationship with each other, their families, communities, and broader society (Leahy et al., 2002; Bekker and Clark, 2016; Kerr and Stirling, 2019).

To date, it can be said that attention has been paid to making sport more equitable on the basis of sex, sexual orientation and disability. Some attention has also been paid to the rights of the child and in particular, the girl child. These are the beginnings of the considerations of intersectionality, or the interconnectedness of social categories (e.g., sex, race, class, disability) which “create overlapping and interdependent systems of discrimination or disadvantage (Cooper, 2016).” Risk of rights’ violations increases where individuals or groups fall into multiple categories.

In general, research on violence against athletes in sport has focused on four groups: the child athlete, the racialized athlete, the Lesbian, Gay, Bisexual, Trans, Queer and Two Spirited (LGBT) athlete, and athletes with disabilities. Particular issues emerge for the girl child, and even more precisely, for the elite child athlete and the elite girl child athlete (Mountjoy et al., 2017; Rhind and Owusu-Sekyere, 2017). In the International Safeguards for Children in Sport, a supporting document shows the critical intersection of aspiring athletes who were refugees, some with disabilities. In 2016, the IOC accepted 10 athletes to compete under the Olympic flag on the first ever 2016 IOC Refugee Olympic Team. Ten were supported through the 2020 Games as are another 40 through the Refugee Athletes Scholarship Program. So too, the IPC formed an Independent Paralympic Team of two Para athletes who competed under the Paralympic flag at the Rio Games. There has been little research with other vulnerable groups such racialized participants, participants with mental health challenges, or those who are disadvantaged in some way economically, regionally, linguistically, or on the basis of religion.

While we do not yet have much research on the specific issues of human rights, disability and abuse, potential exists for research in Para sport on abuse and intersectionality: violence against the child athlete, the girl or boy child athlete, the racial/ethnic minority athlete, the LGBT athlete and so on. Initial research addressing interpersonal violence against Para athletes has not to our knowledge included *also* belonging to a minority group, racial discrimination, being indigenous and being a sexual or ethnic minority.

Just as Para athletes are not a uniform category, disability may or may not inform an individual’s dominant identity(ies). Intersectionality allows for overlapping of gender, race/ethnicity, socioeconomic status, sexuality, disability, etc. as both interrelated and differentially weighted in an individual’s lived experience. As a conceptual framework, intersectionality offers a lens through which researchers can uncover the experiences and meanings of interpersonal violence against diverse Para athletes.

#### Who Is Vulnerable to Human Rights Violations in Sport?

**Figure 1:**
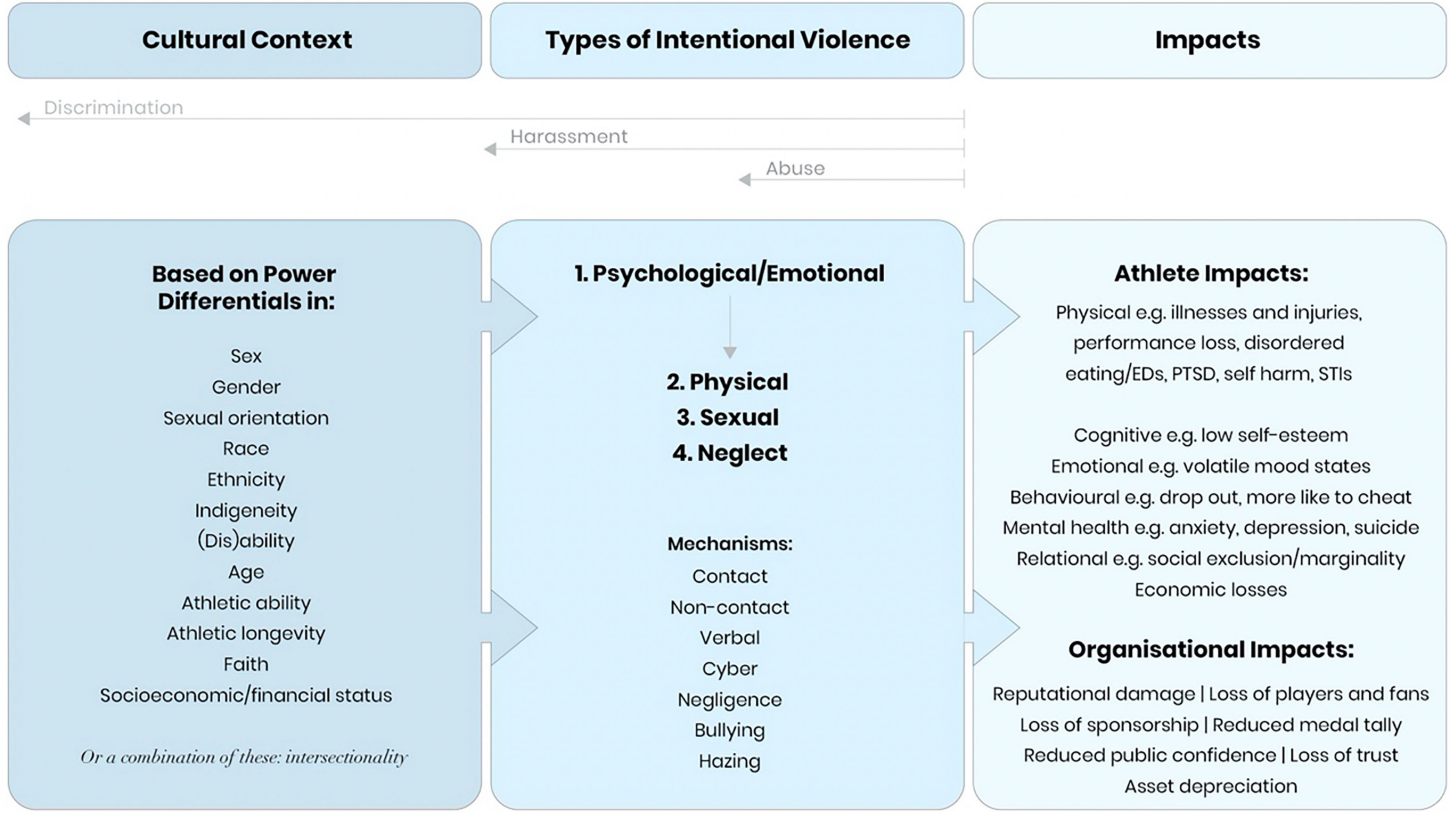
Conceptual model of intentional violence in sport, highlighting the central role a psychological power imbalance plays for all forms of harassment and abuse. Eds, Eating disorders; PTSD, post-traumatic stress disorder; STIs, Sexually transmitted illnesses. Figure adapted from Mountjoy et al. (2016).

All who participate in sport, and particularly those with disabilities are vulnerable to human rights violations in sport. With accessible, actionable, and enforceable human rights protections in place, discrimination and abuse may be less likely. Here we take the lead from Mountjoy et al. on the types of rights abuses (discrimination, harassment, and abuse) athletes may experience (Figure 1), often along one or more of the following lines: sex, gender, sexual orientation, race/ethnicity, indigeneity, (dis)ability, age, athletic ability/status and athletic longevity (Mountjoy et al., 2016). These roughly coincide with the human rights considerations previously described. Relatedly, Brackenridge in her early work identified athlete risk variables (i.e., characteristics) that correlated with increased risk of sexual violence: sex (female), age (younger), size/physique (smaller/weaker), level of awareness of sexual harassment (low), rank or status (potentially high), self-esteem (low), history of sexual abuse in family (unknown or none), relationship with parents (weak), education and training on sexual harassment and abuse (none), medical problems, especially disordered eating (medium/high), dependence on coach (total), devotion to coach (complete), win-at-all-costs mindset, and the “Stage of Imminent Achievement” relative to puberty (at or before; Brackenridge and Kirby, 1997; Brackenridge et al., 2001).

Early on, Brackenridge did not consider disability a risk variable. Almost two decades later, The International Safeguards for Children in Sport, seen by some as the central document for safeguarding all participants in sport from human rights violations, only mentions disability in a supporting document dealing with young refugees (International Safeguards for Children in Sport, 2012). However, the 2016 IOC Consensus Statement contains a section on athletes with disabilities that highlights specific vulnerabilities of Para participants including “(Rhind et al., 2021) making uninformed assumptions about the care needs of athletes, (Hartill et al., 2021) exploiting the athletes’ dependence on personal care (e.g., communication requirements, travel requirements and competition logistics), and (Kerr et al., 2019a) blurring of the roles and responsibilities in the coach–athlete relationship, and, where present, (Vertommen et al., 2016) the caregiver–athlete relationship (Valenti-Hein and Schwartz, 1995; Kirby et al., 2008; Tuakli-Wosornu et al., 2020).” Another factor increasing risk of abuse is the pervasiveness of enabler-bystanders, who become complicit with the institution in, despite either directly witnessing or learning about athlete abuse, chose to remain silent rather than respond to the needs of imperiled athletes (Guiora, 2020).

**Figure 2:**
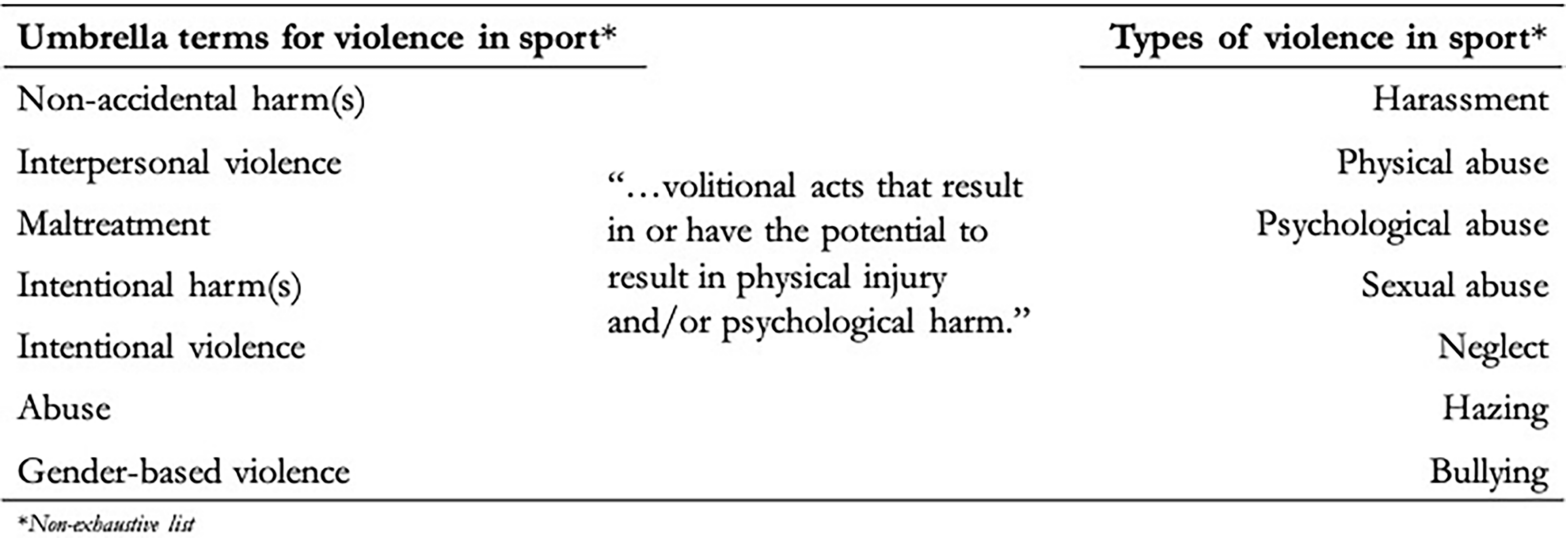
Terminology of harassment and abuse sportspeople may experience.

We use the term ‘interpersonal violence’ to refer to harassment and abuse sportspersons may experience in sport (Figure 2). This is distinct from other forms of violence that may occur on and off the field of play as part of sport. The term ‘non-accidental violence/harm (s)’ has also surfaced on occasion but is out of phase with violence prevention scholarship in non-sport sectors (Dahlberg and Krug, 2006). Both ‘interpersonal violence’ and the less-synchronous term ‘non-accidental violence/harm(s)’ are used elsewhere in sports scholarship (Mountjoy et al., 2016, 2017; Fortier et al., 2020; Tuakli-Wosornu et al., 2020).

Sport safeguarding research to date has focused on children, the nature of and risk factors for abuse, high-performance athletes, national statistics, comparing sport and non-sport rates of abuse, coach-athlete relationships, boys, sexual violence, concealment, and more recently on prevention and cross-vulnerability to abuses across intersecting identities (Kirby et al., 2000; Nielsen, 2001; Demers, 2006; Raakman et al., 2010; Hartill, 2013, 2014 Vertommen et al., 2013; Fasting, 2017; Hartill et al., 2021). This work is increasingly being informed by the voices of athletes with abuse experiences (Viseras, 2015).

#### The Four Main Types of Abuse

The four major forms of interpersonal violence in sport are psychological, physical, sexual, and neglect. Stirling and Kerr and the IOC consensus statement writers agree, that psychological harassment and abuse is the portal through which abusers enter (Mountjoy et al., 2016; Stirling and Kerr, 2016; Kerr and Stirling, 2019). Harassment and abuse athletes may experience is first and foremost psychological, because a real or perceived power imbalance lies at the core of all abuse (Figure 1). It includes “denigrating, belittling or humiliating an athlete, ignoring them, isolating them, shouting or swearing at them, denying them attention and support, and scapegoating them (Leahy et al., 2004; Mountjoy et al., 2015, 2017).” It has been established that when emotional abuse comes from an authority figure, such as a coach or physician, the impact of the negative comments and actions is heightened (Mountjoy et al., 2017). The psychological abuse of Para sport participants follows the same trajectory, though with disability contexts considered, the abuse may be experienced differently (Rutland et al., 2022).

Physical abuse and forced physical exertion, the second form, includes both physical contact abuses such as slapping, punching, tripping, shaking and kicking and use of excessive force, as well as physical maltreatments such as over training, over exertion, forced exertion, under-nourishment, under-hydration, forced consumption (specific foods, drugs and/or alcohol) and poorly advised medical interventions (Brackenridge et al., 2001, 2010b; Leahy et al., 2002; Raakman et al., 2010; Alexander et al., 2011; Mountjoy et al., 2017). Fortier et al. in 2020 put the emphasis on athlete harms rather than on the nature of the physical aggression (Fortier et al., 2020). This could well be a fruitful avenue for further research with Para athletes and their experiences of interpersonal violence. There is no reason to assume patterns of harm are the same for athletes living with and without a disability.

Sexual abuse, the third form, is an umbrella term covering sexual and gender-based harassment and abuse. Sexual and gender-based harassment refers to behaviour that makes an athlete feel sexualized or discriminated against on the basis of gender (or perceived gender). This can be verbal or non-verbal and/or physical or non-physical (Mountjoy et al., 2017). Sexual and gender-based harassment is, as above, a manipulation of power and/or trust. It does not matter whether the abuser intends to harass or abuse an athlete, it is the way in which the athlete feels about the behaviour that is determinant. If it hurts them, it is wrong. Sexual abuse is an abuse of power that involves “sexual activity, with or without penetration, where consent is not or cannot be given, often involving manipulation by grooming and entrapment of the athlete, and sometimes involving aggressive coercion (Brackenridge and Fasting, 2002; Mountjoy et al., 2017).” There is more yet to be learned about the intersection of vulnerabilities of Para participants and sexual and gender-based abuse.

Neglect, the fourth form of interpersonal violence, is the failure to provide what an athlete needs to participate fully and safely in sport training and competition. This includes failure to provide social and physical supports needed for athlete safety and security (Fortier et al., 2020). Examples include: refusing athletes hydration in extreme heat or long practices; refusing needed nutrition both during an sport event and over the long term; making athletes do workouts too heavy and/or too long before they are either physically prepared or physically developed to do so; failure to provide for or withholding of safe equipment and workouts and competitions so that athletes get equipment-failure or overuse injuries; and failure to provide or secure medical attention when indicated (Mountjoy et al., 2017). One might also add, failure to fully consider or disregarding the impact of disabilities when setting up equipment, facilities, training sessions and competition venues (Rutland et al., 2022). What works for non-disabled athletes may not work for certain Para athletes.

Prevalence data that included Para participants is difficult to find. Early on, Para athletes were generally invisible as research participants or, in the case of the Kirby et al. (2000) study, their data were confounded with experiences of athletes who had sustained acute or chronic injuries--an early opportunity lost. Alexander et al. (2011) Vertommen et al. (2016) Kerr et al. (2019a) underlined the size the problem for athletes, and for athletes with disabilities. The rates of psychological abuse range from 37.6 to 75%; for physical abuse from 11.3 to 24%; and for sexual abuse/harm from 14.3 to 32%. Neglect was measured only by Kerr et al. and they found 66% of athletes had experiences with neglect in sport.

Three studies addressed athletes’ lifetime experiences of sexual violence: Leahy et al. (2002) with Australian athletes, Ohlert et al. (2018) with German athletes and Vertommen et al. (2016) with Dutch and Belgian athletes. These studies were large scale surveys, the latter two of which were able to probe athletes’ perception of abuse severity. Ohlert et al. (2018) sent 6,699 questionnaires to German Olympic athletes and 300 to Para national team athletes. Just over 25% responded. The average age of athlete-responders was 21.6 years. Of those, 37.6% experienced at least one sexual violence situation during their lifetime; 11.2% indicated experiencing severe sexual violence. The authors found no significant differences on the basis of dis−/ability. Vertommen et al. (2016) surveyed 4,000+ adults who had played sport as children. 38% reported experiences of psychological violence, 11% physical violence and 14% sexual violence. Also “ethnic minority, lesbian/gay/bisexual (LGB) and disabled athletes and those competing at the international level report significantly more experiences of interpersonal violence in sport.”

This study also captured the severity of the interpersonal violence experiences (mild, moderate, severe). As the authors state, the most important outcome of the 2016 study is the intersectionality of the types of violence and those who experience it (Vertommen, 2018). Athletes who were in the disabled category report 49.7% psychological violence, 32.4% physical violence and 33.4% sexual violence. These compare with and are higher than the average across all respondents of 38.3, 11.9 and 14%. Athletes in racial/ethnic minority subgroups report 39.1, 19.1 and 20.3% respectively, also higher than the average across all respondents. LGBT athletes reported 42, 19.5 and 25.1% respectively; athletes competing internationally disclosed 55, 25 and 28.6%, respectively. Athletes with disabilities had the second highest percentage of psychological abuse and the highest percentages of physical and sexual abuse. The authors did not report more on how many athletes were disabled *and also* international competitors, ethnic minorities, or LGBT.

#### Organizational Drivers of Abuse

It is important to ask how sport culture itself is a risk; and who is at greatest risk (in and out of sport; Roberts et al., 2020). Organizational lethargy, hesitation in accepting the reality of abuse, and/or resistance to change are issues. Violence in sport work has always been difficult for those in sport to accept partly because sport long had a global reputation as a good, healthy, safe and productive place for all. Even today, not all international sport federations (IFs) and national sport federations (NFs) have *any* systems and structures to protect participants and respond to complaints that arise. Many sport organizations have difficulty even discussing abuse.

One of the established facts is that elite athletes experience higher levels of interpersonal violence than non-elite (Mountjoy et al., 2016). Athletes are put at increased risk if the sport is strongly oriented towards winning at any cost, risk-taking, and pushing boundaries (e.g., not correcting “grey zone’ discriminations and behaviours; Vertommen et al., 2016; Roberts et al., 2020). They may comply with requests to travel too often, without adequate medical and social supports, to train when they do not feel safe or well or to meet demands that may injure them or put them at risk of violence.

Mountjoy et al. write that organizational threats to child athletes are the following: abuse from spectators, discrimination, cultures which normalize abuse, unhealthy training programmes, hazing, medical mismanagement, systematic doping and age-cheating (Mountjoy et al., 2015). Denison and Kitchen echo the risks to lesbian and gay athletes because of abuse from spectators and cultures which normalize abuse of these groups (Denison and Kitchen, 2015). They add that lesbian and gay athletes are most at risk in the locker rooms and also in the stands where spectators are located.

A final organizational driver is what Brackenridge called “mission tension” and the appearance of cross-sector partnerships (Brackenridge, 2017). For example, human rights can be developed *through* sport: that is, sport is used as a vehicle through which international development can occur. So too, sport and child protections can be brought together to make progress on social justice issues and children’s rights. This would be sport *with* child protections. For athlete protections, Brackenridge argues that *within* sport is the most appropriate way to protect athletes because there is no ‘mission creep’ from partners. When the *MeToo* and *Black Lives Matter* movements began, sport needed to address the relationship between social activism and sport (Tuakli-Wosornu et al., 2021). They both have an impact on safety and security issues for athletes. Sport has always adapted to “getting it right,” albeit slowly, especially with inclusion issues and now with activism linked with prevention of harm to athletes (Brackenridge, 2017). Para sport is caught up in the same sweeping social justice challenges. We propose activists and sports people work together to get sport right.

Organizations must include in Codes of Conduct and similar policies, that all participant-members have the right “to participate in a non-violent, safe and respectful environment,” and acknowledge their duty of care to ensure that happens (Mountjoy et al., 2016). Beyond the abuser-predator, all sport organizations and actors within those organizations are equally implicated and responsible for fostering safe playing environments (Kerr et al., 2019b). Organizations that abandon athletes in peril are effectively protecting abusive institutions rather than the people to whom duties are owed (Guiora, 2020).

#### Individual-Level Drivers of Abuse

The individual drivers of abuse are integrated into the culture of sports. Aside from individuals with motives to abuse athletes, there are unique sport-specific circumstances which allow individuals more latitude in their behaviours – and possibly, more room to abuse without being suspected and caught, or without being reported. These include unique access points where athletes are inherently vulnerable: having deep involvement in athletes’ sport and personal lives, having unquestioned authority over athletes, being a person in authority who is involved with athletes’ health and well-being, lack of structured scrutiny or oversight, lack of overall supervision; and reduced or no scrutiny when athletes are successful (a rise in impunity; Kirby et al., 2000; Brackenridge et al., 2001; Stirling and Kerr, 2009). These circumstances can be organizationally managed to reduce risk of harm being perpetrated.

For sport in general, data to date suggest perpetrators of abuse are often peer athletes, but mostly male, older coaches of younger athletes, and in positions of authority (coaches, physicians, teachers, instructors; Kirby et al., 2000; Kirby and Wintrup, 2002; Brackenridge et al., 2008, 2010a). Within Para sport, nothing must be taken for granted. Many individuals comprise Para athletes’ entourage – there are at times quite a number of “hands on deck.” These people all should have legitimate reasons for their presence and should have roles to play that are appropriate to their training and professional skills, but also to athletes’ real (rather than assumed) needs. The nature of their interpersonal relationships with athletes must always be able to withstand scrutiny by others. Whether they are coaches, trainers, medical personnel, technicians, sport psychologists and so on, these individuals should all have passed through rigorous screening before being granted access to Para athletes. Family and friends are in a different category than the professionals but are every bit as important to athletes’ wellbeing. If those interpersonal relationships are healthy and supportive, then the athlete benefits. If, however, those interpersonal relationships are difficult, fraught with tensions, and/or exploitative, the wellbeing of the athlete may not be enhanced or worse, may be threatened in their presence.

Other individual drivers have to do with lack of control over ‘grey zone’ behaviours. Individuals with poor personal boundary controls may rely, unfairly and unwisely, on athletes to set the interpersonal boundaries. Boundary-setting is the responsibility of the person who is in the coaching/teaching and/or decision-making position relative to the athlete. This is particularly important when athletes are children (under 18 years; Vertommen and Parent, 2020). When such behaviours are brought to the sports organization’s attention, they can draw the ‘offenders’ attention to poor boundary setting practices (e.g., sexualized joking with athletes) and help them make better decisions to reduce grey zone behaviours. In this way, adults can be coached/trained towards more appropriate behaviours. For this to work, *all* in the sport environment have to know it is their shared responsibility to identify and draw attention to unwanted or suspicious behaviours.

Inaction by individual enablers and bystanders is another powerful driver of abuse. The fact of the matter is, bystanders (actors in the sport environment who are present during a situation, and remain inactive) and enablers (actors in the sport environment who are not present during a situation, but know or should have known and decide to minimize and/or blatantly reject knowledge of harm) are agents of institutional complicity, and are just as implicated as are abusers and sports organizations, in allowing abuse to continue (Guiora, 2020). Time and again, we see that when athletes first disclose abuse, there are always others who knew or ought to have known they were in peril. Unfortunately, most organizational policies are written in such a way that policies spring into action once a disclosure is made. This narrow over-reliance on grievance reporting is firstly based on fallacious common-sense assumptions (i.e., the assumptions that abuse will be recognized, and that the aggrieved will overcome the forces in sport that actively work to suppress disclosure). Secondly, in tethering safeguarding policies and remedies to reporting systems, sports organizations place the burden squarely and unfairly on the shoulders of athletes, when it should be on all sport actors. There are always many who know, but do not say, do not report, and do not act.

Finally, peer athletes can drive abuse (Mountjoy et al., 2016; Kerr et al., 2019a,b). Team pressure may play a role in abused (e.g., bullying, hazing and initiations). Other motivators may include opportunity, desire, and need to have power over another (Brackenridge et al., 2001). We are just beginning to scratch the surface on peer abuse in sport and very little is available on peer abuse and Para sport (Tuakli-Wosornu et al., 2020).

## Discussion

All forms of interpersonal violence, including harassment, bullying, hazing, disability stigma, physical, psychological, and sexual abuse, neglect, as well as gender- and race-based discrimination, constitute human rights violations. It is clear that abuse in sport and in Para sport is prevalent, generally tolerated and relative to its scope and impact, under-examined. It is also clear that those in Para sport present a powerful example of how sport for all can be done with integrity and by ethical means. Inclusion and equity are important human rights drivers for all of sport, and Para sport has shown interesting ways in which these can be achieved. One example is the staggered start system in Para Triathlon, where athletes from more than one sport class participate in the same medal event and have an equal chance to win.

First, from the interpersonal violence data on Para athletes, the creation of preventative strategies and remedy pathways that specifically consider the complex needs of Para athletes may provide the same for other athletes. This is not to say there are one-size-fits-all safeguarding strategies – on the contrary, safeguarding strategies conceived of globally must be interpreted and applied locally (if applied at all), in specific cultural contexts (Rutland et al., 2022). However, as athletes with potential social vulnerabilities, including disabilities, gender non-conformity and minority racial/ethnic identities may have the highest degree of vulnerability to abuse, they may represent the archetypal population of concern. Despite this, the overwhelming majority of published scholarship about athletes with disabilities focuses on the positive outcomes of participation, without concomitant acknowledgement of the interpersonal risks (Martin, 2013; Wright et al., 2019). Further, due to the limited amount of data, the prevalence and impact of abuse in Para athletes remains unclear and information on trends over time is similarly unavailable. A central question at the heart of the matter is: why are the athlete groups for whom protections are most urgently needed, also the cohorts for whom rigorous evidence is most scarce? Is this a case of denial (or another unconscious defensive distortion of reality) because the perceived truth is too challenging for individuals and institutions to handle (Vaillant, 1994)? What are the implications of this literature gap for the quality, relevance, and generalizability of currently available data – and what could be gained by closing it?

Second, working within the Para sport context means that lower-income status must come to the forefront. This would then allow for the lower-income reality of many athletes, including non-Para athletes, to be considered in policy and planning, and in reporting of violence pathways. This is because 80% of those with disabilities globally live in low- and middle-income environments, and there is compounded risk of interpersonal violence for low-income Para athletes (World Health Organization, 2011). In some ways, Para athletes from lower-income settings represent the most relatable group for ‘universal’ protections. This is because whilst safeguarding policies and programs developed for higher-needs athletes in lower-resource settings may be able to function effectively for athletes with greater independence in more highly-resourced settings, the inverse is not true: safeguarding policies and programs developed for relatively independent athletes in higher-income settings may not work in more challenging environments, and are therefore neither inclusive nor global.

Third, Para sport provides a perfect opportunity to address the impact of abuse, in all its complexities, on all athletes through their life-course. If we can understand, in a comprehensive way, the Para sport participants’ abuse impacts, we are then well prepared to understand those of non-Para athletes as well. The cascade of downstream athlete impacts following abuse (e.g., death by suicide, sport drop-out, unintentional injury, and decreased performance) can be catastrophic (Mountjoy et al., 2016; Vertommen et al., 2018). Thus the rationale for the *prevention* of intentional injury in Para sport settings is clear, but the empirical data related to incidence, prevalence, and risk factors for various modes of abuse in Para sport, as well as contextualized patterns of abusive behaviours, and the impact of cultural and geographical setting that preventative and therapeutic strategies rely on are absent. Importantly, we wish to emphasize here that one cannot assume identical manifestations and similar athlete-level perceptions of abuse exist in Para and non-disabled sport environments – a one-size-fits-all approach may not work, considering the fixed disparities in social privilege those with and without disabilities experience (Patatas et al., 2018).

Here, privilege matters (Angell, 1993; Brown and White, 2020). Privilege impact wellbeing and influences risk of negative health outcomes such as violence and other forms of personal harm. For simplicity, privilege refers to “a right, benefit, advantage, or opportunity.” Privilege has recently been identified as one among myriad social determinants of health (SDOH), i.e., “the conditions in which people are born, grow, work, live, and age, and the wider set of forces and systems shaping the conditions of daily life (Witten and Maskarinec, 2015; Brown and White, 2020)” A socially constructed value system based on society’s perception of human life, privilege – like all SDOH – can positively impact longevity, and diminish risk of health-related pathology. Generally, people of high socioeconomic status, racial majorities, those without disabilities, and those who identify as heteronormative are advantaged relative to other groups. These favorable social conditions positively impact mental and physical health outcomes over time. All other variables being held equal, athletes without disabilities can be considered privileged relative to those who are disabled. As a protective force, privilege limits the amount and degree of untoward health exposures the privileged group experiences.

Safeguarding data from athletes of a wide range of impairments would be applicable to and beneficial for any athlete group that is equally or less vulnerable to interpersonal violence, i.e., those that have proportionately more social privilege. Bringing Para sport into the safeguarding debate may therefore increase its global relevance and impact. Beyond the inclusion of Para sport, there is an opportunity to seek guidance and leadership from it, as Para sport actors may at least equally be apt to understand nuanced systems of oppression.

Fourth, if we do not attend to the Para sport experience, the human rights promise of equity and inclusion in sport go for naught. While, as McNamee says, “Para sport is not a purity beacon,” it does show how sport can be an evolving living experience as it continually changes and adapts through its mistakes and its triumphs. Including the Para sport challenges with classification of athletes provides incredible examples of what is needed to make meaningful, equitable and inclusive participation possible for all sport (McNamee and Parry, 2021). In the same way that examining unconscious bias, implicit (gender and/or race) bias, and microaggressions without due consideration and involvement of minority groups risks missing important content, omitting Para sport from the safeguarding canon may extinguish opportunities for contextualized strategies and solutions forged in experience to be brought to the fore. Including Para athletes’ voices, in their own words, at their own paces, and from their own environments, may enable us to uncover athlete-centered, athlete-generated understandings of – and strategies to dismantle – the harrowing reality for victimized athletes of all backgrounds and abilities.

## Conclusion

Consideration for athletes’ human rights is part of Para sport’s origin story. As all forms of interpersonal violence against athletes are rights violations, we may be able to strengthen abuse prevention and athlete protection measures by looking to the Para and similar sports movements. Inevitably, within Para and similar sport settings, there will occasionally be misguided decisions that are antithetical to a rights-based ethos. But these do not condemn the innate ideals of the Para movement on the whole. These ideals are what the sports world is challenged to focus on in the present analysis.

A re-interpretation of the violence prevalence studies with an eye to intersectionality is warranted. It moves us from seeing Para athletes (or any participant in sport at any level) as single-category participants (e.g., female athlete, etc.) into seeing them as whole people navigating complex worlds of intersecting characteristics and identities (e.g., female, elite, disabled and racialized, for example). It should move us towards an applied understanding of interpersonal violence in sport, and make the analysis of rights violations more pragmatic. This requires us to be more open-minded, nimble and nuanced researchers, and requires all in sport to reach for different rules of engagement based on respect, equity, safety and a ‘person first, athletes second’ paradigm (Campbell and Tincknell-Smith, 2021).

**Figure 3:**
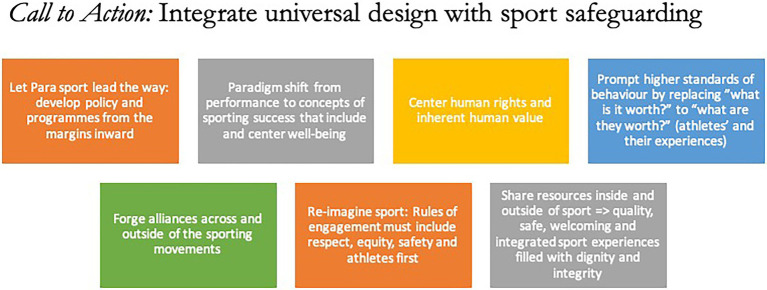
Proposed action steps to integrate universal design with sport safegaurding (non-exhaustive list).

Centering athletes’ rights and repositioning Para sport as leader, may help pave the path forward in athlete safeguarding (Figure 3). All athletes are human beings first. As such, their fundamental value and self-worth go far beyond their (exhilarating) performances in the realm of sport. There may be no sporting movment that understands this more genuinely than the Paralympic Movement and similar initiatives. For all who are committed to athlete protections, achieving sports environments that are free from harassment and abuse in a way that goes beyond theories and can be truly applied, is about fundamentally changing one’s mindset – starting with the questions we ask ourselves during training and competitions, as we interact with teammates, coaches, trainers, clinicians, and others, and decide how to behave.

In toxic win-at-all-costs high-performance sport cultures, many ask, “what is IT worth?” (performance, profit, acclaim, admiration). This can easily lead to unethical behaviours (i.e., doping, cheating, toxicity, abuse) in the blind pursuit of external validators of sporting success, in an environment where the end is believed to justify any and all means. When the underlying assumption is shifted away from notions of athletes as (replaceable) commodities whose value is tethered only to performance, and towards an awareness of athletes’ inherent value as full human beings worthy of respect and dignity, the question becomes “what are THEY worth?” (self, team, sport). Importantly, these two concepts are not in competition. The best athletic performances are often produced in the most responsible sports environments. The fact is, when athletes are in full health, mentally, physically, and socially, performances flourish. This philosophy rejects the capitalist approach to sport and takes a humane stance.

This philosophy is exactly where the Paralympic Movement started ideologically – indeed, it was a simple but radical paradigm shift, away from performance and towards respecting human dignity and inclusion, that sparked and continues to sustain global disability sport at all levels. Para sport in its original form provides a guidepost for a global commitment to creating sports environments where dignity and integrity are amplified rather than sacrificed in the name of performance, and where “success” itself is defined by rights-based notions of human achievement through holistic growth and development. Of all sports movements, the Paralympic and similar movements have perhaps the greatest imperative to accurately reflect the best of humanity – from grit and resilience to fundamental decency. Leveraging these ideals may help close the gaps in the safeguarding foundation, and create a new, higher standard of athlete protections that all of sport can benefit from.

## Author Contributions

YT-W and SK conceptualized and designed this manuscript, drafted the initial manuscript, and reviewed and revised the manuscript. All authors contributed to the article and approved the submitted version.

## Conflict of Interest

The authors declare that the research was conducted in the absence of any commercial or financial relationships that could be construed as a potential conflict of interest.

## Publisher’s Note

All claims expressed in this article are solely those of the authors and do not necessarily represent those of their affiliated organizations, or those of the publisher, the editors and the reviewers. Any product that may be evaluated in this article, or claim that may be made by its manufacturer, is not guaranteed or endorsed by the publisher.

## Footnotes

^1^Person-first language has been more commonly adopted in peer-reviewed writing since the mid-1970’s, but it may inadvertently reinforce disability stigma by identifying impairments as fundamental deficits. Identity-first language on the other hand, historically rebuffed in the academy, may reinforce a disability-positive viewpoint, where impairments are woven into people’s core identity, inherent value, and sense of belonging.
